# P-1394. TB Exposed: Understanding the Risks and Patient Factors Associated with Healthcare Worker Exposure to Pulmonary Tuberculosis at a Major Medical Center

**DOI:** 10.1093/ofid/ofaf695.1581

**Published:** 2026-01-11

**Authors:** Lucy B Tran, Meghan Madhusudan, Wanda H Jones, Sharon E Fawcett, Jonathan Grein, Michael Ben-Aderet

**Affiliations:** UCLA Multicampus Fellowship in Infectious Diseases, Los Angeles, CA; Cedars Sinai Medical Center, Los Angeles, California; Cedars Sinai Medical Center - Los Angeles, CA, Los Angeles, California; Cedars Sinai Medical Center, Los Angeles, California; Cedars-Sinai Medical Center, Los Angeles, CA; Cedars Sinai Medical Center, Los Angeles, California

## Abstract

**Background:**

Tuberculosis (TB) remains a leading global cause of death from a single infectious agent with rising cases annually. Health care workers (HCWs) are at increased risk of TB - up to three times higher than the general population, though this varies across studies. In this study, we reviewed data from a single US hospital to examine patient factors associated with HCW exposure, HCW data, and interferon-gamma release assay (IGRA) conversion rates.

**Methods:**

We conducted a retrospective case series from January 2018 to July 2024 at a 915 bed tertiary-care teaching hospital in Los Angeles, California. All patients with a positive pulmonary culture or molecular test for *Mycobacterium tuberculosis* obtained during a hospital admission were included. Patients associated with HCW exposure were compared to those without using student’s t-test for continuous variables and Fisher’s exact test for categorical variables. Exposures were determined by the hospital epidemiology department using Los Angeles County Department of Public Health criteria and HCW data was obtained from departmental records.

**Results:**

There were 64 pulmonary TB cases identified of which 20 resulted in exposures to 230 HCWs. The average age of patients associated with HCW exposure was 65 years, compared with 54 years (p=0.011) for patients not associated. Patients associated with exposure were more likely to be foreign-born (95% vs 70%, p=0.047), and had a longer duration before their first TB diagnostic test (6.6 vs 2.5 days, p=0.017). We also observed a trend in increased HCW exposure among patients admitted without respiratory conditions (60% vs 34%, p=0.0618) and those without ID consult (70% vs 89%, p=0.084). HCW most associated with exposures were nurses (50%), nursing assistants (19%), and physicians (17%). A median of 8 HCWs were exposed per patient, ranging from 3 to 45 HCWs. Eight HCWs were exposed to >1 TB patient. There were no IGRA conversions among exposed HCWs.

**Conclusion:**

Our study showed pulmonary TB patients associated with HCW exposure were older, more likely foreign-born, and had delayed recognition possibly due to admissions for non-respiratory conditions. Remarkably, no IGRA conversions occurred among exposed HCWs, suggesting the occupational risk of TB transmission in modern hospital setting is low.

**Disclosures:**

All Authors: No reported disclosures

